# Staging and Tumor Characteristics of Human Papillomavirus (HPV)-Associated Oropharyngeal Cancer in Northern Nevada: A Retrospective Descriptive Study

**DOI:** 10.7759/cureus.95625

**Published:** 2025-10-28

**Authors:** Steven T Cho, Samantha Chang, Nicholas Bazett, Sydney Althoff, Stefan Harpster, Nadya Vinsdata, Dylan Wrye, Katharine Thomas

**Affiliations:** 1 Hematology and Oncology, School of Medicine, University of Nevada, Reno, Reno, USA; 2 Hematology and Oncology, Renown Health, Reno, USA

**Keywords:** health equity, hpv, hpv vaccination, nevada, opscc, p16

## Abstract

Human papillomavirus (HPV)-associated oropharyngeal squamous cell carcinoma (OPSCC) has demonstrated demographic and geographic disparities in national datasets, but regional data remain limited. This retrospective study analyzed 296 OPSCC patients diagnosed or treated at Renown Regional Medical Center in Northern Nevada between 2014 and 2024. Clinical and demographic variables, including sex, county of residence, and Nevada income quintile, were compared with tumor stage, size, nodal involvement, and metastasis using the chi-squared tests. Stage IV disease was most common at diagnosis (36.5%; N=108), and the majority of patients were male (79.7%; N=235) and White (94.9%; N=281). No statistically significant associations were found between staging variables and sex (p=0.05), geography (p=0.73), or income level (p=0.532). These findings suggest potential regional equity in staging at diagnosis and highlight the need for expanded studies that incorporate broader social determinants and multicenter data to validate trends in HPV-mediated OPSCC presentation.

## Introduction

Oropharyngeal squamous cell carcinoma (OPSCC) represents the most common malignancy of the oral cavity, accounting for over 90% of oral cancers worldwide [1]. A significant subset of these are driven by human papillomavirus (HPV) infection, with p16 overexpression serving as a reliable surrogate marker and prognostic tool for HPV-related disease [2,3]. Previous trends show that the incidence of HPV-driven OPSCC has steadily risen [4]. While national data provide valuable insights into the epidemiology of HPV-positive OPSCC, substantial geographic and demographic variability remains. Understanding these regional differences is critical for tailoring prevention, screening, and treatment efforts to the populations most at risk.

Studies have shown that lower socioeconomic status is associated with later-stage diagnosis and worse survival outcomes, partly due to disparities in healthcare access and screening utilization [5,6]. Semprini et al. found that rural populations are more likely to present with advanced disease and have limited access to specialized oncologic care [7]. Bharmjeet and Das found that symptom presentation and patterns of metastasis can vary based on age, sex, race, and socioeconomic background [8]. Furthermore, Lebo et al. found that lower household income was independently associated with higher tumor stage at presentation and delayed time to treatment initiation [9]. Others found that individuals living in medically underserved or rural areas were less likely to receive guideline-concordant care, which has a direct impact on survival [10,11]. In a national Surveillance, Epidemiology, and End Results (SEER) database analysis, Gaubatz et al. found that geographic disparities persist even after controlling for insurance and socioeconomic status, suggesting that location itself, through proximity to care, provider availability, and public health infrastructure, plays an independent role in outcomes [12]. As such, comprehensive regional analyses are essential to identify at-risk subgroups, guide early detection strategies, and inform public health interventions tailored to the unique needs of specific communities.

To address gaps in regional demographic knowledge of HPV-mediated OPSCC, this study aimed to explore the demographic and clinical characteristics of p16-positive OPSCC patients in Northern Nevada. By focusing on this specific population, we seek to identify local trends and disparities that may differ from national data, thereby informing targeted prevention, screening, and treatment strategies tailored to the needs of this community.

## Materials and methods

The study protocol was reviewed by the Institutional Review Board (IRB) of the University of Nevada, Reno, who granted a waiver of informed consent for a retrospective study of deidentified data. A list of all OPSCC patients diagnosed or treated at Renown Regional Medical Center (RRMC) between 2000 and 2024 was then attained through the Pennington Cancer Institute's Cancer Registry. Patients who did not have p16 testing or had cancers other than in the oropharynx were excluded from the study. An electronic medical record (EMR) was then used to collect information from the remaining patients. Collected parameters include clinical characteristics (tumor location, tumor, node, metastasis (TNM) and staging per the American Joint Committee on Cancer (AJCC) 8th Edition, tumor size, and p16 testing results) and demographical information (age, sex, county of residence, race, ethnicity, and residential zip code). Patients who had any of the above information missing from their EMR were excluded from the study.

Self-reported zip codes were used to determine patients' county of residence. Their socioeconomic status were estimated using their county of residence combined with mean income data from the US Census. Five-year estimates of county-level Nevada income data, reflecting average values from 2018 to 2022, were obtained from data.census.gov using the most recent 2023 release. County-level mean household income was extracted from Table S1901 "Income in the Past 12-Months (in 2023 Inflation-Adjusted Dollars)", while income quintile distributions for Nevada counties were obtained from Table B19081 "Mean Household Income of Quintiles". Patients were then sorted into income quintiles using their reported county of residence, with quintiles 1 and 5 representing the lowest and highest socioeconomic quintiles, respectively. Income data was unavailable for Nye and Humboldt counties, and patients from those counties were excluded from economic variable analyses. Logistic regression models assessed associations between p16 positivity and the selected variables. All analyses were performed using the Stata 18.5 Basic Edition software (StataCorp., College Station, TX, USA). Results were reported as odds ratios (ORs) with 95% confidence intervals (CIs) and corresponding p-values, with statistical significance defined as p<0.05. Associations between p16 status and these categorical variables were evaluated using the chi-squared (χ²) tests. Statistical significance was defined as p<0.05.

## Results

A total of 296 patients diagnosed with oropharyngeal carcinoma were included in this study. The majority were male (79.7%; N=236) and White (94.9%; N=281), with most residing in Washoe county (72%; N=213). Washoe is home to Nevada's second-largest city and is also the only county in Northern Nevada to have an urban classification. Socioeconomically, the highest proportion of patients fell within the third (59.2%; N=170) and fourth (31.7%; N=91) Nevada income quintiles; notably, no patients were from the highest (fifth) quintile. At the time of diagnosis, stage IV disease was most common (36.5%; N=108), followed by stage I (26.4%; N=78), stage III (19.3%; N=57), and stage II (17.9%; N=53). In terms of tumor size, T2 (35.5%; N=105) and T3 (27%; N=80) were most frequently observed. Lymph node involvement was common, particularly N1 (36.8%; N=109) and N2 (34.5%; N=102), while M1 was present in 10% (N=29) of cases (Table 1).

**Table 1:** Cancer staging and population demographics Income data was unavailable for Nye and Humboldt counties. Patients residing in these counties (3%; N=9) were excluded from economic analyses leading to a smaller N.

Staging		N (%)
Staging	I	78 (26.4%)
	II	53 (17.9%)
	III	57 (19.3%)
	IV	108 (36.5%)
Tumor size	T0	12 (4.1%)
	T1	60 (20.27%)
	T2	105 (35.5%)
	T3	80 (27%)
	T4	39 (13.2%)
Lymph node status	N0	53 (17.9%)
	N1	109 (36.8%)
	N2	102 (34.5%)
	N3	31 (10.5%)
	N4	1 (0.3%)
Metastasis	M0	267 (90.2%)
	M1	29 (10%)
Demographics		N (%)
Sex	Male	236 (79.7%)
	Female	60 (20.3%)
County	Washoe	213 (72%)
	Other	83 (28%)
Race	White	281 (94.9%)
	Asian	3 (1%)
	Black	2 (0.68%)
	Hispanic	1 (0.34%)
	Indigenous	7 (2.4%)
	Other	2 (0.68%)
Nevada income quintile (N=287)	1st ($23,700)	2 (0.7%)
	2nd ($42,400)	24 (8.4%)
	3rd ($53,100)	170 (59.2%)
	4th ($65,500)	91 (31.7%)
	5th ($101,500)	0 (0%)

Sex-based comparisons revealed that males were more likely to present with stage IV disease compared to females (88 vs. 20), while females showed relatively higher rates of stage III and T4 tumors. Although the association between sex and stage at diagnosis approached statistical significance (p=0.05), no significant differences were observed for tumor size (p=0.07), lymph node status (p=0.11), or metastasis (p=0.36) by sex. Geographic comparisons between Washoe county and other counties showed no significant variation in stage (p=0.73), tumor size (p=0.17), nodal status (p=0.17), or presence of metastasis (p=0.71). Similarly, no significant associations were found between cancer characteristics and socioeconomic status as measured by Nevada income quintiles. For instance, while stage IV disease was most prevalent in the third and fourth quintiles, the differences in staging (p=0.532), tumor size (p=0.618), lymph node status (p=0.42), and metastasis (p=0.70) were not statistically significant across income levels (Table 2).

**Table 2:** All analyzed demographical and socioeconomic variables were not associated with tumor stage at the time of diagnosis All values were written as N (%); statistical significance was defined as p<0.05. Income data was unavailable for Nye and Humboldt counties. Patients residing in these counties (3%; N=9) were excluded from economic analyses leading to a smaller N. OR: odds ratio; CI: confidence intervals

Staging								
Sex	Staging	Male	Female	χ² value				P-value
	I	62 (21%)	16 (5%)	7.70				0.05
	II	47 (16%)	6 (2%)					
	III	39 (13%)	18 (6%)					
	IV	88 (30%)	20 (7%)					
County	Staging	Washoe	Other counties	χ² value				P-value
	I	57 (19%)	21 (7%)	1.30				0.73
	II	41 (14%)	12 (4%)					
	III	39 (13%)	18 (6%)					
	IV	76 (26%)	32 (11%)					
Nevada income quintile (N=287)	Staging	1st	2nd	3rd	4th	5th	χ² value	P-value
	I	0 (0%)	8 (3%)	42 (14%)	25 (8%)	0 (0%)	8.02	0.532
	II	0 (0%)	2 (0.6%)	35 (12%)	14 (5%)	0 (0%)		
	III	0 (0%)	3 (1%)	35 (12%)	17 (6%)	0 (0%)		
	IV	2 (0.6%)	11 (4%)	58 (20%)	35 (12%)	0 (0%)		
Tumor size								
Sex	Tumor size	Male	Female	χ² value				P-value
	T0	11 (4%)	1 (0.03%)	8.80				0.07
	T1	45 (15%)	15 (5%)					
	T2	84 (28%)	21 (7%)					
	T3	70 (24%)	10 (3%)					
	T4	26 (9%)	13 (4%)					
County	Tumor size	Washoe	Other counties	χ² value				P-value
	T0	7 (2%)	5 (2%)	6.40				0.17
	T1	48 (16%)	12 (4%)					
	T2	77 (26%)	28 (9%)					
	T3	58 (20%)	22 (7%)					
	T4	23 (8%)	16 (5%)					
Nevada income quintile (N=287)	Tumor size	1st	2nd	3rd	4th	5th	χ² value	P-value
	T0	0 (0%)	1 (0.3%)	9 (3%)	2 (0.6%)	0 (0%)	9.97	0.618
	T1	0 (0%)	4 (1%)	33 (12%)	23 (8%)	0 (0%)		
	T2	1 (0.3%)	5 (2%)	58 (20%)	37 (13%)	0 (0%)		
	T3	1 (0.3%)	10 (3%)	46 (16%)	19 (7%)	0 (0%)		
	T4	0 (0%)	4 (1%)	24 (8%)	10 (4%)	0 (0%)		
Lymph node status								
Sex	Lymph node status	Male	Female	χ² value				P-value
	N0	38 (13%)	15 (5%)	7.60				0.11
	N1	88 (30%)	21 (7%)					
	N2	86 (29%)	16 (6%)					
	N3	23 (8%)	7 (2%)					
	N4	0 (0%)	1 (0.3%)					
County	Lymph node status	Washoe	Other counties	χ² value				P-value
	N0	7 (2%)	5 (2%)	2.67				0.17
	N1	48 (16%)	12 (4%)					
	N2	77 (26%)	28 (9%)					
	N3	58 (20%)	22 (7%)					
	N4	23 (8%)	16 (5%)					
Nevada income quintile	Lymph node status	1st	2nd	3rd	4th	5th	χ² value	P-value
	N0	0 (0%)	3 (1%)	37 (13%)	11 (4%)	0 (0%)	12.35	0.42
	N1	0 (0%)	10 (3%)	59 (21%)	36 (13%)	0 (0%)		
	N2	1 (0.3%)	10 (3%)	52 (18%)	36 (13%)	0 (0%)		
	N3	1 (0.3%)	1 (0.3%)	21 (7%)	8 (3%)	0 (0%)		
	N4	0 (0%)	0 (0%)	1 (0.3%)	0 (0%)	0 (0%)		
Metastasis								
Sex	Metastasis	Male	Female	χ² value				P-value
	Absent	210 (71%)	56 (19%)	0.85				0.36
	Present	25 (9%)	4 (1%)					
County	Metastasis	Washoe	Other counties	χ² value				P-value
	Absent	193 (65%)	74 (25%)	0.14				0.71
	Present	20 (7%)	9 (3%)					
Nevada income quintile	Metastasis	1st	2nd	3rd	4th	5th	χ² value	P-value
	Absent	2 (0.6%)	22 (8%)	150 (52%)	84 (29%)	0 (0%)	0.70	0.70
	Present	0 (0%)	2 (0.6%)	20 (7%)	7 (2%)	0 (0%)		

## Discussion

In this retrospective cohort of 296 OPSCC patients from Northern Nevada, we observed that the majority were White males in middle-income brackets, with a high frequency (36.5%; N=108) of advanced (stage IV) disease at presentation, consistent with known patterns in HPV-mediated disease [1,2,13]. We found no statistically significant associations between disease stage or tumor burden and either socioeconomic status or geographic location. Previous studies on the impact of geographic location on OPSCC outcomes have conflicting conclusions [14-18]. This highlights the need for further investigation into the impact of the location of residence on cancer outcomes. Meanwhile, our unremarkable socioeconomic findings conflict with national data demonstrating notable disparities in outcomes based on socioeconomic and racial factors [5-13]. For example, prior analyses have shown that lower socioeconomic status is associated with later-stage presentation, decreased likelihood of receiving timely or guideline-concordant treatment, and worse overall survival in OPSCC and other pharyngeal cancers [5,8-10]. Lebo et al. found that household income independently predicted the stage at diagnosis, with lower income linked to more advanced disease [9]. Similarly, Semprini et al. reported that rural and socioeconomically disadvantaged patients were less likely to receive appropriate oncologic care and had inferior outcomes, even when controlling for clinical variables [7]. Minas et al. further emphasized the role of systemic barriers in care access, citing disparities in early detection and treatment related to income, geography, and insurance coverage [11]. 

The absence of significant socioeconomic or geographic associations in our cohort may reflect regional strengths in healthcare access, particularly at our tertiary center, or sample-specific characteristics, such as the predominance of patients from Washoe county. However, our study may be underpowered to detect subtle differences, given the limited representation of the extremes of the socioeconomic status distribution (e.g., no patients in the highest income quintile) and relatively small numbers in non-Washoe regions. 

We also observed a marginally non-significant higher rate of stage IV disease among males (p=0.05), which aligns with national and international literature showing male predominance in HPV-mediated OPSCC and a higher nodal burden at presentation [1,2,8]. These findings emphasize the importance of considering sex-based disparities in prevention strategies, including HPV vaccination and early detection campaigns. Racial disparities, though well-documented nationally [8,12], could not be meaningfully assessed in our study due to the overwhelmingly White demographic of the cohort (94.9%; N=281).

Our findings highlight the heterogeneous nature of OPSCC presentation and suggest that regional healthcare delivery plays a critical role in mitigating disparities. As emphasized by Gaubatz et al., geographic location remains an independent predictor of outcome, with structural barriers such as travel distance, specialist access, and public health infrastructure significantly impacting care quality and survival [12]. Although we did not observe statistically significant variation by region or income in our cohort, these findings underscore the need for continued surveillance and investment in regional oncology infrastructure.

As a single-center retrospective analysis, our findings may not be fully generalizable to other regions or healthcare systems. The absence of HPV DNA testing is another limitation, although p16 testing remains a validated surrogate marker in clinical practice [19]. Furthermore, we lacked data on several potentially relevant social determinants of health, such as education level, occupational exposure, and marital status. HPV vaccination status is another important consideration for OPSCC that was notably missing from our study. Since its first release in 2006, the HPV vaccine has emerged as the gold standard preventative for HPV-related cancers. Analyzing how social determinants of health affect HPV vaccine uptake will be especially important for future studies with rising rates of vaccine hesitancy and as the first cohort of vaccine recipients reaches adulthood. These variables may offer additional insight into the mechanisms underlying sex-based HPV transmission disparities.

## Conclusions

This study offers a regional analysis of HPV-associated OPSCC, demonstrating a predominance of male and White patients presenting with advanced-stage disease. However, no significant associations were found between cancer stage and either income level or geographic location. These findings may reflect strengths in local healthcare access but also underscore the need for more detailed investigation into the social determinants that influence cancer presentation. Future studies should incorporate molecular HPV testing, expanded sociodemographic data, and multicenter cohorts to better capture regional and national trends. Ongoing efforts to enhance early detection, increase HPV vaccination uptake, and ensure equitable access to care across socioeconomic groups will be critical to addressing the growing burden of HPV-related oropharyngeal cancer.
